# The anxiolytic effect of dexmedetomidine in adult perioperative patients: a systematic review with meta-analysis of randomized controlled trials

**DOI:** 10.3389/fpsyt.2026.1957118

**Published:** 2026-09-15

**Authors:** Kai Yang, Ji-Zheng Cui, Wen-Juan Wang, Jia-Xuan Yang

**Affiliations:** 1 Department of Anesthesiology, The Second Affiliated Hospital of Soochow University, Suzhou, China; 2 Department of Pain, Lianyungang Maternal and Child Health Hospital Affiliated to Kangda College of Nanjing Medical University, Lianyungang, China; 3 Pain Management Department, Suzhou TCM Hospital Affiliated to Nanjing University of Chinese Medicine, Suzhou, China

**Keywords:** dexmedetomidine, locus coeruleus, meta-analysis, noradrenergic system, perioperative anxiety, postoperative recovery, sleep quality

## Abstract

**Background:**

Perioperative anxiety in adults is common and impairs postoperative recovery. The use of dexmedetomidine during perioperative procedures is increasing, but evidence on its effects on anxiety-related outcomes remains to be systematically synthesized.

**Methods:**

This systematic review was based on searches of PubMed, Embase, Web of Science, MEDLINE, and the Cochrane Library from database inception through May 4, 2026. Eligible studies were randomized controlled trials evaluating perioperative dexmedetomidine in adults and reporting quantitative anxiety outcomes.

**Results:**

Twelve RCTs involving 1,373 participants were included. Overall anxiety scores were lower with dexmedetomidine than with control (SMD = −0.73; 95% CI [−1.37, −0.10]; P = 0.028; I² = 94.2%; 95% prediction interval [−2.97, 1.50]). Within 24 h after surgery, the pooled estimate likewise favored dexmedetomidine (6 RCTs; SMD = −0.83; 95% CI [−1.58, −0.07]; P = 0.038; I² = 92.9%; 95% prediction interval [−2.76, 1.11]). The estimates for intraoperative anxiety, anxiety at 24–72 h postoperatively, and anxiety beyond 72 h were SMDs = 0.08 (95% CI [−2.31, 2.46]), −0.11 (95% CI [−0.41, 0.20]), and −0.31 (95% CI [−1.02, 0.39]), respectively. Postoperative sleep quality favored dexmedetomidine (4 RCTs; SMD = −0.64; 95% CI [−0.81, −0.48]; I² = 0%), whereas no clear between-group differences were observed for depression or pain.

**Conclusion:**

Perioperative dexmedetomidine was associated with reduced anxiety in adult surgical patients. Although the evidence for this association was clearer within 24 h after surgery, the limited data across time windows do not establish that the anxiolytic effect is confined to this period.

**Systematic review registration:**

https://www.crd.york.ac.uk/PROSPERO/, identifier CRD420251129414.

## 1 Introduction

Perioperative anxiety is not a single emotional response but rather a state of physical and mental tension arising from multiple stressors such as surgery and anesthesia (1). Anxiety can intensify the body’s stress response (e.g., sympathetic nervous system activation, increased norepinephrine and cortisol secretion), which may exacerbate postoperative pain and impede the recovery process (1, 2). Previous systematic reviews and meta-analyses have shown that preoperative anxiety is common among surgical patients, with a pooled global prevalence of approximately 48% (3). Higher levels of preoperative anxiety have been associated with several adverse postoperative outcomes, including poorer postoperative pain control, greater analgesic requirements, and an increased risk of postoperative delirium (3, 4). Therefore, controlling perioperative anxiety may help improve postoperative recovery and enhance overall patient outcomes.

Dexmedetomidine is a highly selective α_2_-adrenergic receptor agonist that acts at α_2_-adrenergic receptors in the locus coeruleus of the pons (5). It inhibits norepinephrine release through negative feedback, producing natural sleep-like sedation and anxiolytic effects (5, 6). Dexmedetomidine is currently used clinically for short-term sedation in intubated and mechanically ventilated patients, as well as an adjunct for perioperative anesthetic sedation. Compared with traditional benzodiazepine sedatives, dexmedetomidine is characterized by a well-defined sedative profile, ease of arousal, and minimal respiratory depression (6, 7). In recent years, dexmedetomidine has garnered significant attention for its use in perioperative sedation, with its pharmacological actions positioning it as a potential agent for alleviating perioperative anxiety (1, 8–10).

Research on the anti-anxiety effects of dexmedetomidine during the perioperative period is growing, and its impact on outcomes such as perioperative anxiety is receiving increasing attention. A multicenter prospective study reported that intraoperative dexmedetomidine administration was associated with reduced postoperative anxiety symptom incidence in elderly patients undergoing non-cardiac surgery (11). Recent randomized controlled trials (RCTs) in cardiac surgery also demonstrated significantly lower postoperative anxiety rates in the dexmedetomidine group compared to placebo (12). Collectively, these studies suggest that dexmedetomidine has short-term anxiolytic potential in perioperative patients. However, the dosage, timing of administration, comparator type, and patient populations varied across studies, and hemodynamic adverse events, particularly bradycardia, remain an important safety concern (13). In addition, some studies found no significant effects of dexmedetomidine on anxiety, depression, or other psychological outcomes at 30 days after surgery, suggesting that its effects may not persist over the longer term (12).

Although dexmedetomidine has received increasing attention for its potential anxiolytic effects, anxiety has generally been examined only as a secondary outcome within broader assessments of postoperative recovery or neuropsychiatric outcomes. A systematic synthesis specifically evaluating the anxiolytic efficacy of dexmedetomidine in adult perioperative patients is still lacking. To address this gap, we conducted a systematic review and meta-analysis to evaluate the effects of dexmedetomidine on perioperative anxiety in adults. We also examined postoperative sleep quality, depression, and pain as secondary outcomes to provide a broader assessment of its perioperative effects.

## 2 Materials and methods

This systematic review complied with the Preferred Reporting Items for Systematic Reviews and Meta-Analyses (PRISMA) guidelines (14). The protocol was registered in PROSPERO (CRD420251129414). Detailed methods are provided in Supplementary Material.

### 2.1 Literature search

PubMed, Embase, Web of Science, MEDLINE, and the Cochrane Library were searched from database inception to May 4, 2026. Keywords and free-text terms were combined; the full search strategy is detailed in Supplementary Material S1. We also traced references from included studies and relevant reviews. Two researchers independently screened abstracts and full texts; disagreements were resolved by a third reviewer.

### 2.2 Inclusion and exclusion criteria

Inclusion: Adult (≥18 years) perioperative RCTs; intervention involving dexmedetomidine administration at any perioperative time point/route; comparator comprising standard care, placebo, or conventional sedation or anxiolysis strategies (benzodiazepines, propofol, gabapentinoids, esketamine, etc.); reporting of quantifiable anxiety scales. Exclusion: Pediatric or age-unclear studies; non-English-language publications; combined interventions where dexmedetomidine effects cannot be disentangled; non-randomized designs or studies with unobtainable data. Study characteristics and potential effect modifiers (region, age, anesthesia type, surgery type, baseline anxiety, dexmedetomidine dose/route/timing, control category, etc.) were extracted using a standardized form. Authors were contacted for missing data when necessary.

The primary outcome was anxiety score. For the primary anxiety analysis, one clinically relevant eligible assessment was selected from each independent comparison to avoid including the same participants more than once. Time-specific meta-analyses were categorized as intraoperative, within 24 h postoperatively, 24–72 h postoperatively, and beyond 72 h postoperatively. When a study reported multiple assessments within the same time window, the latest assessment was used. Secondary outcomes included postoperative sleep quality, depression, and pain. Safety outcomes were summarized narratively when differences in outcome definitions or insufficient reporting precluded quantitative synthesis.

### 2.3 Data extraction and management of complex designs

Two reviewers independently extracted study characteristics, participant numbers, anesthesia and surgery type, dexmedetomidine route and regimen, comparator category, measurement instrument, assessment time, and outcome data. Effect directions were harmonized across outcomes so that negative values consistently indicated lower anxiety, depression, and pain scores and better sleep quality with dexmedetomidine. When continuous data were reported as medians and interquartile ranges, means and standard deviations were estimated using established methods, and the influence of these conversions was evaluated in sensitivity analyses (15).

For trials with multiple eligible groups, clinically similar arms were combined using Cochrane methods. When independent comparisons did not share participants, they were retained as separate analytic units. For subgroup analyses in which a shared arm contributed to more than one comparison, its sample size was divided across comparisons while its mean and standard deviation were unchanged, thereby preventing double counting and preserving the appropriate unit of analysis (16).

### 2.4 Risk of bias and certainty of evidence

Two reviewers independently assessed each included RCT using the Cochrane Risk of Bias 2 (RoB 2) tool. Disagreements were resolved by discussion or third-reviewer adjudication. Certainty of evidence was evaluated using GRADE across risk of bias, inconsistency, indirectness, imprecision, and publication bias (16).

### 2.5 Statistical analysis

Statistical analyses were performed using R version 4.5.3. For outcomes measured using different scales, effect estimates were expressed as Hedges’ g standardized mean differences (SMDs); when the same scale was used, mean differences (MDs) were calculated. All effect estimates were reported with 95% confidence intervals (CIs). Because differences across studies were anticipated in patient populations, surgical procedures, dexmedetomidine regimens, comparators, and outcome measures, all meta-analyses used a prespecified random-effects model. Between-study variance (τ²) was estimated using restricted maximum likelihood, and 95% CIs for pooled estimates were adjusted using the Hartung–Knapp method to better account for uncertainty when few studies were available (17). When estimable, 95% prediction intervals were calculated to describe the plausible range of true effects in similar clinical settings (18).

Between-study heterogeneity was assessed using Cochran’s Q test and quantified using τ² and I², representing between-study variance and the proportion of variability attributable to heterogeneity, respectively. Prespecified analyses included separate meta-analyses by anxiety assessment time window and subgroup analyses by comparator type. Subgroup analyses by anesthesia type and anxiety measurement instrument were exploratory (16).

Sensitivity analyses included leave-one-out analyses and analyses excluding studies in which means or standard deviations were estimated from medians and interquartile ranges, to assess the robustness of the pooled estimates. For the overall anxiety outcome, small-study effects were explored using a contour-enhanced, sample-size-based funnel plot together with the Pustejovsky–Rodgers test. Conventional Egger regression was not used because standard SMD estimates are mathematically correlated with their standard errors (19). No correction for multiple testing was applied to exploratory subgroup analyses; therefore, these findings are hypothesis-generating only. Two-sided P values <0.05 were considered statistically significant. Results were interpreted by considering the magnitude of effect, confidence and prediction intervals, between-study consistency, and certainty of evidence, rather than statistical significance alone.

## 3 Results

### 3.1 Literature screening and characteristics of included studies

A total of 1,051 records were identified after searching five databases: PubMed, Embase, Web of Science, MEDLINE, and Cochrane (**Figure 1**). Following the removal of 316 duplicate records, 735 records were included for screening. After reviewing titles and abstracts, 707 studies were excluded for the following reasons: 431 were irrelevant records, 153 focused on pediatric populations, and 123 did not report relevant anxiety-related outcomes. The remaining 28 studies underwent full-text review, with 16 excluded because valid quantitative anxiety data could not be extracted (n = 14) (20–33), non-English language (n=1) (34), and protocol (n=1) (35). Ultimately, 12 studies met the criteria and were included in the meta-analysis (**Table 1**) (36–47). Included studies primarily originated from China, India, Iran, Malaysia, and other regions, predominantly involving patients undergoing general surgery, obstetrics and gynecology, or orthopedic procedures. Mean participant age across the included trials ranged from 28 to 69 years. 10 RCTs enrolled physically healthy participants (ASA I-II), while the others included some ASA III patients. All studies administered dexmedetomidine perioperatively (primarily intravenously; dosage and administration details are provided in **Table 1**). Control groups included saline, midazolam, propofol, esketamine, gabapentin, and others. All 12 RCTs reported anxiety scores; among them, 4 studies reported postoperative sleep quality scores, 4 studies reported postoperative depression scores and 4 studies reported postoperative pain scores.

**Figure 1:**
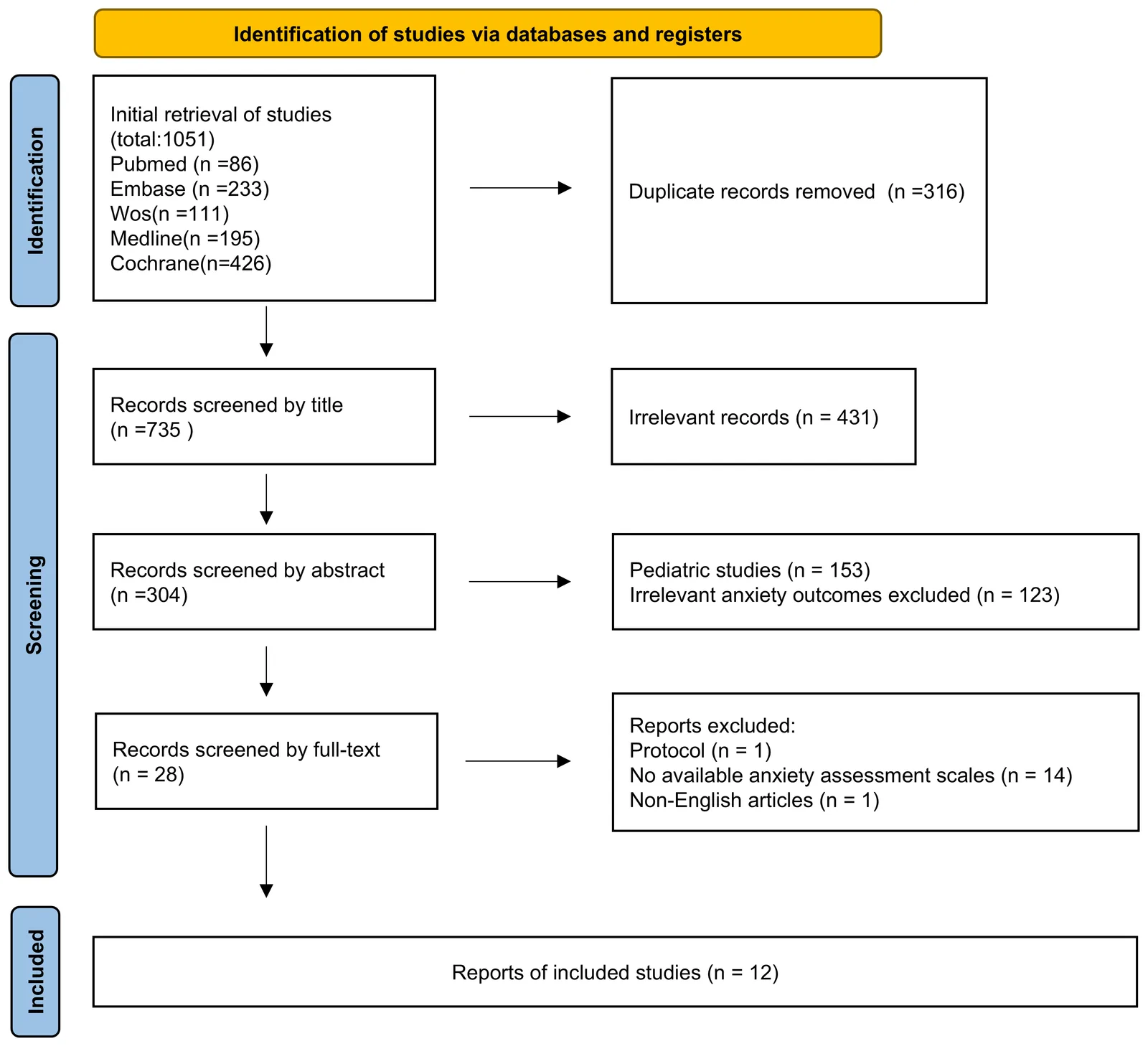
Preferred reporting items for systematic reviews and meta-analyses (PRISMA) flow diagram of study selection.

**Table 1:** Characteristics of studies included in the systematic review and meta-analysis.

Author	Region	Control group	n DEX/control	Mean age DEX/control, y	Anesthesia	Surgery	DEX intervention	Anxiety measure	Assessment timing	Reported follow-up	Other reported outcomes
Jouybar	Iran	Gabapentin/Melatonin	33/33/33	47.58/50.15/48.79	General	Laminectomy	IV 1 μg/kg	HADS	T1	24 h postoperatively	Pain
Kulkarni	India	Midazolam	30/30	28.5/29.1	General	Minor obstetric and gynecologic procedures	IV 1 μg/kg	VAS-A	T1	1 h postoperatively	–
Li	China	Normal saline	60[2]/60[3]	31.8/30.5	Epidural	Cesarean delivery	Epidural 1 μg/mL + DEX 0.5 μg/mL PCEA for 2 d	SAS	T3	42 d postoperatively	Depression; Sleep; Pain
Lim	Malaysia	Propofol	42/42	40.43/40.60	Combined spinal-epidural	Elective and emergency surgery	IV 0.5 μg/kg, then 0.5 μg/kg/h	VAS-A	T0	Until ward transfer	–
Mukherjee	India	Propofol	10/10	42/42	Local anesthesia with moderate sedation	Orbital surgery	IV 1 μg/kg, then 0.2-0.6 μg/kg/h	VAS-A	T0	Until PACU discharge	Pain
Wang	China	Normal saline	60/60	65.6/66.7	General	Ureteroscopic holmium laser lithotripsy	IV 0.4 μg/kg/h	S-AI	T1/T2/T3	72 h postoperatively	–
Zhang	China	Esketamine	35/35	30.1/29.2	Spinal	Cesarean delivery	IV 1 μg/kg	HADS	T2	72 h postoperatively	Depression; Sleep
Zi	China	Propofol	62/61	64.6/66.2	General	Off-pump coronary artery bypass grafting	IV 0.3-1.0 μg/kg/h	SAS	T2	5 d postoperatively	–
Fu	China	Normal saline	31/34	67.21/67.1	General	Ureteroscopic holmium laser lithotripsy	IV 0.4 μg/kg/h	S-AI	T1/T2/T3	72 h postoperatively	Depression
Fu*	China	Normal saline	35/32	66.7/66.9	General	Ureteroscopic holmium laser lithotripsy	IV 0.4 μg/kg/h	S-AI	T1/T2/T3	72 h postoperatively	Depression
Gu	China	Normal saline	30/30	38.8/37.9	General	Functional endoscopic sinus surgery	IV 1 μg/kg	S-AI	T0	30 min after administration	–
Gu*	China	Normal saline	30/30	37.5/38.3	General	Functional endoscopic sinus surgery	IV 1 μg/kg	S-AI	T0	30 min after administration	–
He	China	Alprazolam/Saline	40/40	68.45/69.2	General	Elective laparoscopic gynecologic surgery	Intranasal 1.5 μg/kg	GAD-7	T1/T3	5 d postoperatively	Sleep
Yu	China	Normal saline	156/154	41.2/39.2	General	Emergency trauma surgery	IV 0.1 μg/kg/h	BAI	T1/T2/T3	72 h postoperatively	Pain; Sleep

BAI, Beck Anxiety Inventory; DEX, dexmedetomidine; GAD-7, 7-item Generalized Anxiety Disorder scale; HADS, Hospital Anxiety and Depression Scale; IV, intravenous; PACU, post-anesthesia care unit; PCEA, patient-controlled epidural analgesia; S-AI, State Anxiety Inventory; SAS, Self-Rating Anxiety Scale; VAS-A, visual analogue scale for anxiety. Timing: T0, intraoperative; T1, ≤24 h after surgery; T2, 24–72 h after surgery; T3, >72 h after surgery. For the overall anxiety synthesis, Lim was represented by the assessment 10 min after the start of surgery, and Fu 2024 by the postoperative 8-h assessment. *Separate independent comparison from the same study. The “Other reported outcomes” column describes outcomes reported by each trial and does not imply that every outcome was included in the quantitative synthesis. [Numbers] indicate patients with missing data due to consent withdrawn or refusal of assessments.

The Cochrane Risk of Bias Assessment Tool 2.0 (RoB 2.0) was used to evaluate the methodological quality of the 12 included RCTs. Overall, most studies had low risk of bias in randomization and outcome measurement (**Figures 2**, **3**).

**Figure 2:**
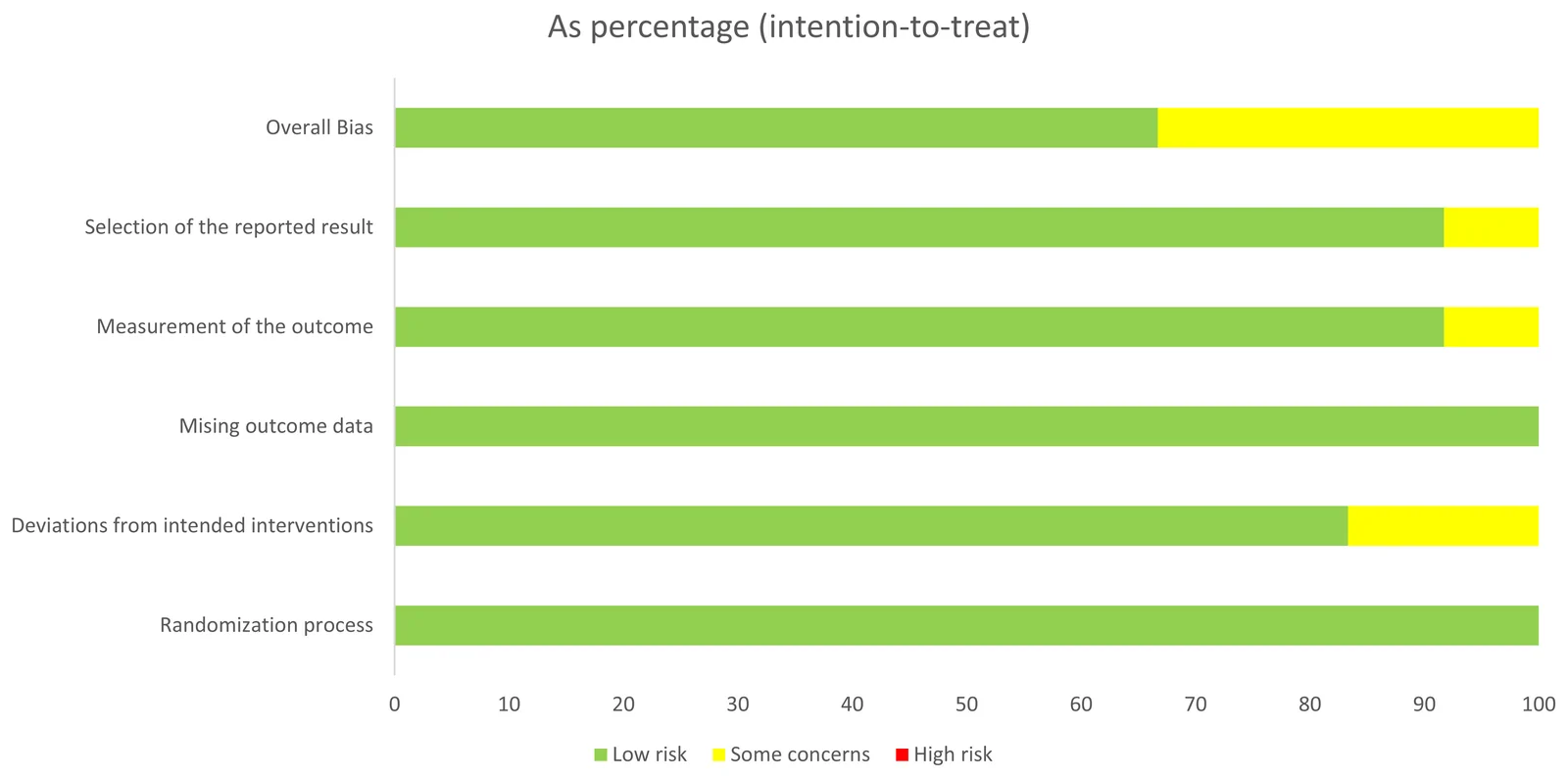
Risk-of-bias graph for the included randomized controlled trials.

**Figure 3:**
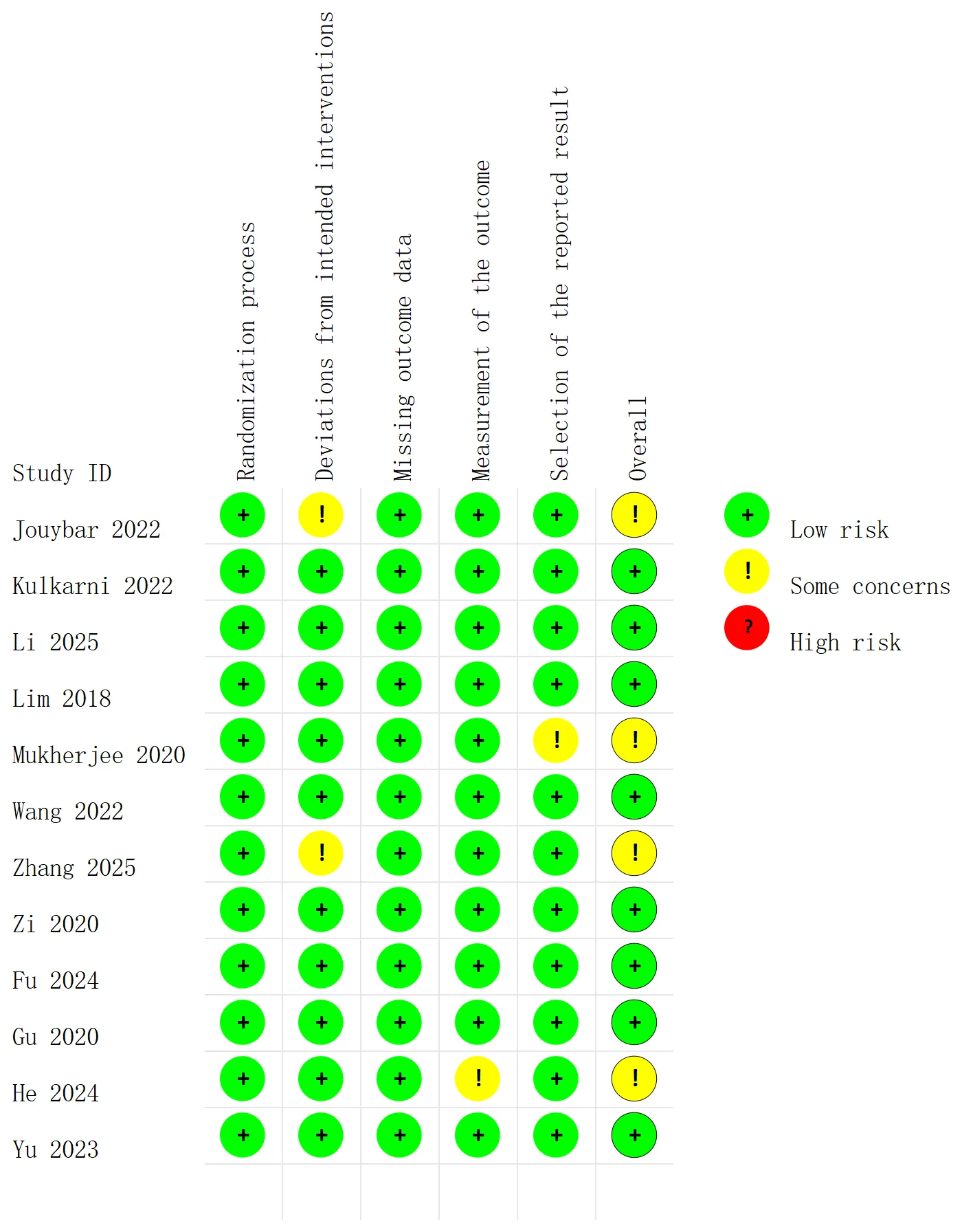
Risk-of-bias summary for the included randomized controlled trials.

### 3.2 Primary outcome

Twelve RCTs involving 1,373 participants were included in the overall anxiety analysis. The random-effects model showed that anxiety scores were lower, on average, with dexmedetomidine than with control (SMD = −0.73; 95% CI [−1.37, −0.10]; P = 0.028; 95% prediction interval [−2.97, 1.50]; **Figure 4**). Between-study heterogeneity was high (τ² = 0.946; I² = 94.2%).

**Figure 4:**
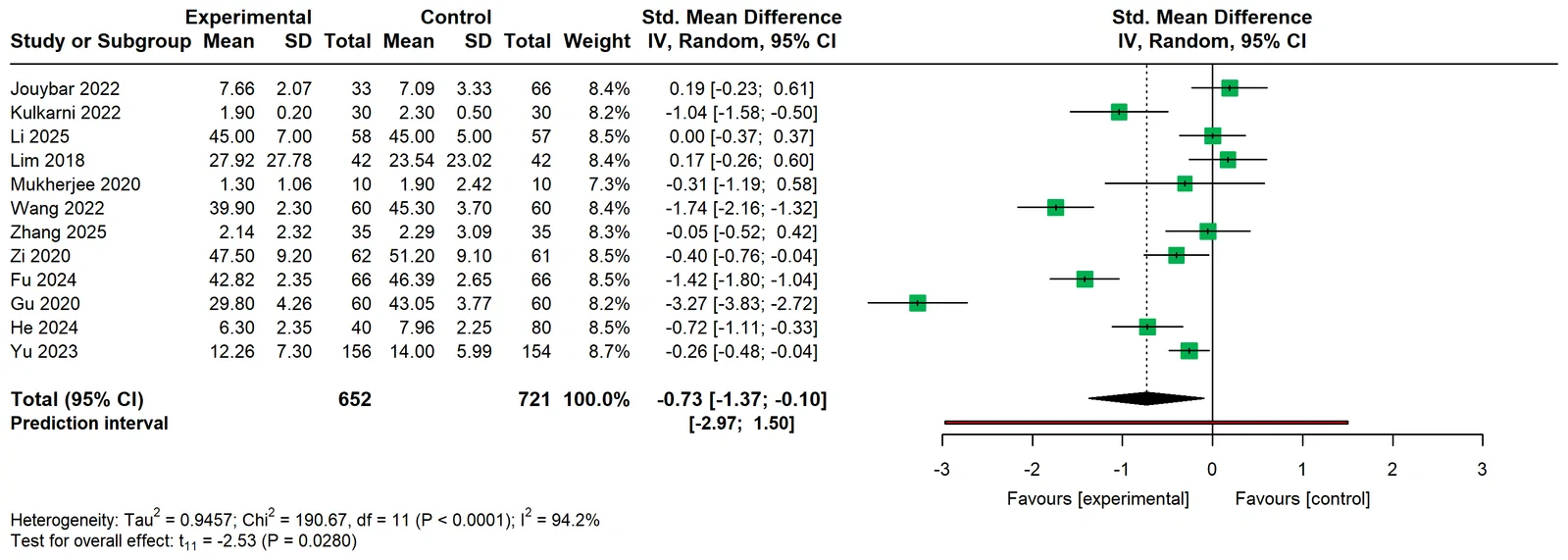
Forest plot of the overall effect of perioperative dexmedetomidine on anxiety scores.

The intraoperative anxiety analysis included 2 RCTs involving 104 participants. The pooled estimate provided no clear evidence of a difference in anxiety scores between the dexmedetomidine and control groups (SMD = 0.08; 95% CI [−2.31, 2.46]; P = 0.746; I² = 0%; **Figure 5**).

**Figure 5:**
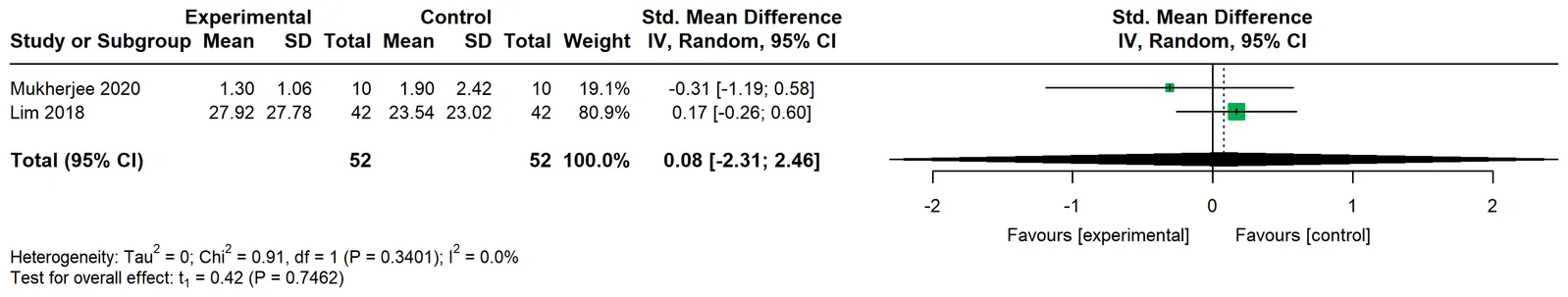
Forest plot of intraoperative anxiety scores.

The analysis within 24 h postoperatively included 6 RCTs involving 841 participants. Anxiety scores were lower, on average, with dexmedetomidine than with control (SMD = −0.83; 95% CI [−1.58, −0.07]; P = 0.038; 95% prediction interval [−2.76, 1.11]; **Figure 6**). However, between-study heterogeneity was high (I² = 92.9%).

**Figure 6:**
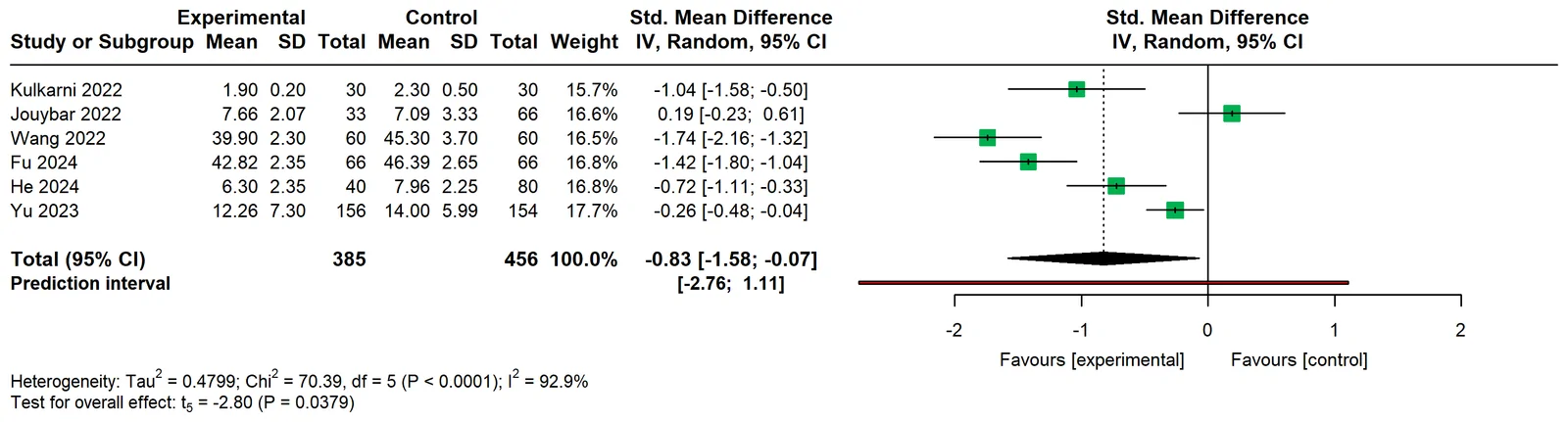
Forest plot of anxiety scores within 24 h after surgery.

The analysis at 24–72 h postoperatively included 5 RCTs involving 752 participants. The pooled estimate was close to the null, and the 95% CI included 0 (SMD = −0.11; 95% CI [−0.41, 0.20]; P = 0.393; I² = 50.8%; 95% prediction interval [−0.69, 0.47]; **Figure 7**). The analysis beyond 72 h postoperatively included 3 RCTs involving 358 participants, and the 95% CI for the pooled estimate likewise included 0 (SMD = −0.31; 95% CI [−1.02, 0.39]; P = 0.195; I² = 55.4%; 95% prediction interval [−1.46, 0.83]; **Figure 8**).

**Figure 7:**
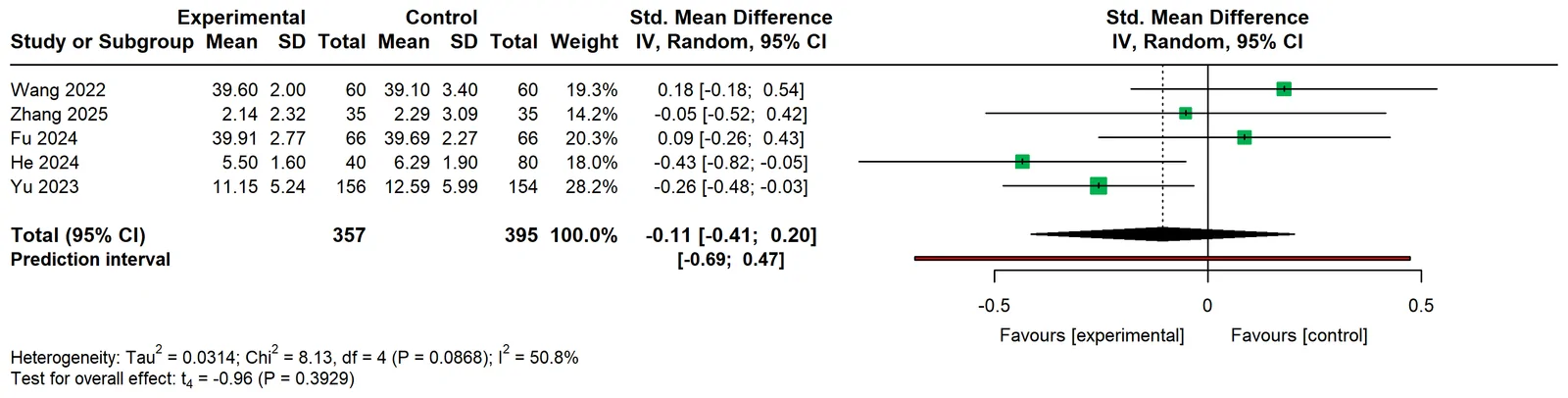
Forest plot of anxiety scores 24–72 h after surgery.

**Figure 8:**
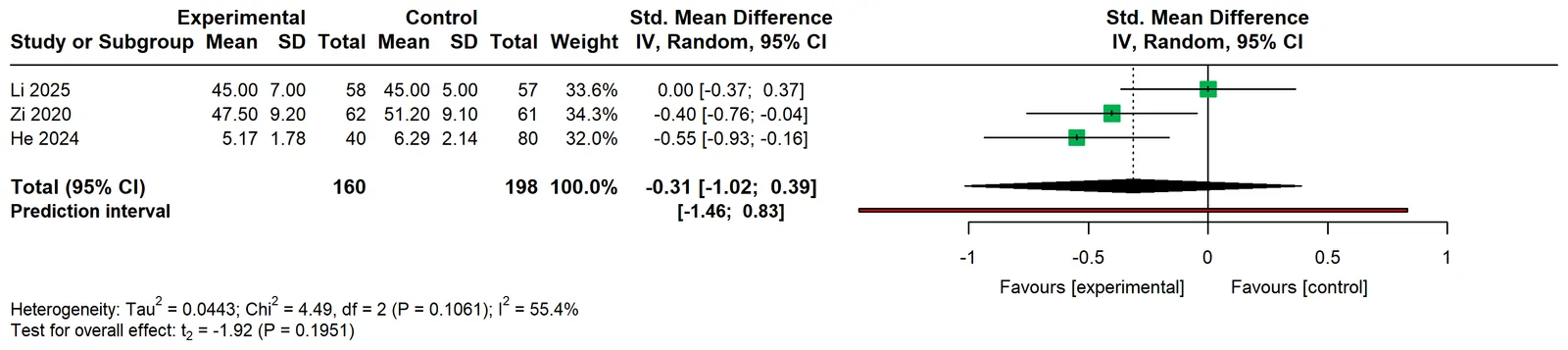
Forest plot of anxiety scores more than 72 h after surgery.

Because some studies reported repeated measurements from the same participants across multiple time windows, the pooled estimates for the different time windows were not independent. Moreover, substantial between-study heterogeneity was observed in both the overall anxiety analysis and the analysis within 24 h after surgery; these pooled estimates should therefore be interpreted cautiously as average effects across heterogeneous clinical settings. Accordingly, the time-window analyses were intended only to describe anxiety effects at different perioperative stages.

The prespecified subgroup analysis by comparator type found no statistical evidence that the pooled effect differed between comparator groups (P for subgroup difference = 0.335; **Supplementary Figure 1**). The subgroup-specific point estimates were in the direction of lower anxiety scores with dexmedetomidine, and the confidence intervals included the null in both comparator subgroups (active comparator: SMD = −0.64; 95% CI [−1.54, 0.26]; saline control: SMD = −1.27; 95% CI [−2.65, 0.11]). Between-study heterogeneity was high in both subgroups (active comparator: I² = 90.3%; saline control: I² = 95.6%).

In a sensitivity analysis excluding saline-controlled comparisons, the available evidence did not establish that dexmedetomidine reduced anxiety compared with active comparator interventions (SMD = −0.64; 95% CI [−1.54, 0.26]; P = 0.138; I² = 90.4%; 95% prediction interval [−3.20, 1.92]; **Supplementary Figure 2**).

The exploratory subgroup analysis by anesthesia type found statistical evidence that pooled effects differed between subgroups (P for subgroup difference = 0.009; **Supplementary Figure 3**). Anxiety scores were lower, on average, with dexmedetomidine in studies using general anesthesia; no statistical evidence of a difference between dexmedetomidine and control was found in studies using non-general anesthesia (general anesthesia: 8 RCTs; SMD = −1.07; 95% CI [−1.97, −0.17]; non-general anesthesia: 4 RCTs; SMD = −0.04; 95% CI [−0.34, 0.27]). Between-study heterogeneity was high in the general-anesthesia subgroup and was not detected in the non–general-anesthesia subgroup (general anesthesia: I² = 95.5%; non-general anesthesia: I² = 0%). The exploratory subgroup analysis by anxiety measurement instrument also found statistical evidence that pooled effects differed between subgroups (P for subgroup difference = 0.006; **Supplementary Figure 4**). Heterogeneity remained high in this analysis (I² = 86.7%).

In the leave-one-out analysis, the point estimate for the overall anxiety effect remained negative after exclusion of any single study. When Wang 2022 or Fu 2024 was excluded, the direction of effect was unchanged, but the 95% CI included 0 (Wang 2022: SMD = −0.64; 95% CI [−1.31, 0.03]; P = 0.059; Fu 2024: SMD = −0.67; 95% CI [−1.36, 0.02]; P = 0.056). Between-study heterogeneity remained high (**Supplementary Figure 8**).

After excluding studies in which means and standard deviations were estimated from medians and interquartile ranges, overall anxiety scores remained lower with dexmedetomidine than with control (9 RCTs; 909 participants; SMD = −0.97; 95% CI [−1.79, −0.15]; P = 0.027; I² = 94.8%; 95% prediction interval [−3.50, 1.56]; **Supplementary Figure 9**). The sensitivity analysis for anxiety within 24 h postoperatively yielded a result in the same direction (SMD = −0.95; 95% CI [−1.87, −0.02]; P = 0.047; I² = 91.8%; **Supplementary Figure 10**). Overall, the sensitivity analyses did not alter the direction of effect; however, the 95% CIs included 0 in some leave-one-out analyses, and substantial between-study heterogeneity persisted.

The contour-enhanced, sample-size-based funnel plot showed no clear pattern of effect size varying with sample size, and the Pustejovsky–Rodgers test likewise detected no evidence of small-study effects (P = 1.00; **Supplementary Figure 18**).

### 3.3 Other perioperative outcomes

Four RCTs involving 437 participants reported postoperative depression scores. The pooled analysis found no statistical evidence of a difference in postoperative depression scores between dexmedetomidine and control (SMD = −0.25; 95% CI [−0.51, 0.00]; P = 0.051; I² = 0%; **Supplementary Figure 5**).

Four RCTs involving 549 participants reported postoperative pain scores. The pooled analysis found no statistical evidence of a difference in postoperative pain scores between dexmedetomidine and control (MD = −1.02; 95% CI [−2.11, 0.08]; P = 0.060; I² = 84.6%; **Supplementary Figure 6**).

Four RCTs involving 620 participants reported postoperative sleep quality. The pooled analysis favored dexmedetomidine, indicating better postoperative sleep quality than with control (SMD = −0.64; 95% CI [−0.81, −0.48]; P = 0.001; I² = 0%; **Supplementary Figure 7**).

Safety outcomes were reported inconsistently and were therefore not quantitatively pooled. Among the trials providing explicit categorical event counts, bradycardia was reported in 34 of 383 patients (8.9%) receiving dexmedetomidine and 23 of 380 patients (6.1%) receiving control interventions, whereas hypotension was reported in 49 of 353 (13.9%) and 44 of 350 (12.6%) patients, respectively. These crude proportions are presented for descriptive context only and should not be interpreted as pooled comparative estimates because event definitions, monitoring periods, and reporting methods differed across trials. Only one trial explicitly reported a bradycardic event requiring pharmacologic intervention: 1 of 30 patients receiving dexmedetomidine required atropine. Another trial prespecified rescue thresholds for bradycardia and hypotension but reported that no patient required rescue treatment. Several other trials reported only serial hemodynamic measurements or qualitative statements without categorical event counts, precluding a reliable estimate of the frequency of clinically significant or intervention-requiring events across all included trials (**Supplementary Table 2**).

## 4 Discussion

### 4.1 Principal findings

This systematic review and meta-analysis found that mean anxiety scores were lower among adult surgical patients receiving perioperative dexmedetomidine than among those in the control groups. In the time-window analyses, the pooled estimate within 24 h postoperatively likewise indicated lower anxiety scores with dexmedetomidine. As a separate secondary outcome, postoperative sleep quality was also better with dexmedetomidine, with no evident between-study heterogeneity. Although the average pooled effects favored dexmedetomidine, between-study heterogeneity was high for both overall anxiety and anxiety within 24 h postoperatively, and the corresponding prediction intervals crossed the null, suggesting that the magnitude of effect may vary across clinical settings. Accordingly, the available evidence suggests that dexmedetomidine may reduce perioperative anxiety and improve postoperative sleep; however, the duration of its anxiolytic effect, the patient populations most likely to benefit, and the optimal treatment regimen remain uncertain.

### 4.2 Potential mechanisms underlying the early anxiolytic findings and improved sleep

The more clearly favorable pooled estimate for anxiety within 24 h postoperatively may be partly explained by dexmedetomidine’s modulation of central noradrenergic activity and the perioperative stress response, although current evidence does not establish that these mechanisms account for the observed temporal pattern. By activating central α_2_-adrenergic receptors in the locus coeruleus, dexmedetomidine produces a sleep-like sedative state from which patients remain readily arousable (13). Previous systematic reviews have also shown that perioperative dexmedetomidine may reduce epinephrine, norepinephrine, and cortisol levels and attenuate certain inflammatory responses (48).

Animal studies have further suggested two potential anxiolytic mechanisms. In a mouse model of acute stress, dexmedetomidine suppressed the excitability of corticotropin-releasing hormone neurons in the hypothalamic paraventricular nucleus through α_2_-adrenergic receptors. In a mouse model of chronic stress, dexmedetomidine attenuated the hyperactivity of noradrenergic neurons in the locus coeruleus and normalized abnormally elevated norepinephrine levels in the medial prefrontal cortex (49, 50). These findings suggest that reduced central arousal and suppression of stress-related neural activity may contribute to the anxiolytic effects of dexmedetomidine.

The anxiolytic effect observed within 24 h may be partially attributable to residual sedation rather than a specific reduction in anxiety pathophysiology. The included studies did not consistently report sedation scores concurrent with anxiety assessments, precluding disentanglement of these effects.

Improved sleep may partly share a biological basis with the noradrenergic modulation described above. Previous perioperative meta-analyses have reported improved subjective sleep quality during the early postoperative period with dexmedetomidine; some polysomnographic studies have also observed increases in sleep efficiency and stage N2 sleep (51). Experimental studies in healthy adults have further suggested that the increase in anxiety induced by sleep deprivation is associated with impaired medial prefrontal cortex activity and disrupted connectivity with limbic regions (52). Thus, reduced anxiety scores and improved sleep quality may both reflect attenuation of central hyperarousal.

Evidence across perioperative time windows was limited: only two studies reported intraoperative anxiety outcomes, relatively few studies reported anxiety outcomes at later postoperative time points, and some trials contributed repeated measurements from the same participants to more than one time window. Because these repeated measurements are correlated, the corresponding pooled estimates across windows did not represent independent replications, and the usual independence assumption therefore does not hold when the estimates are considered jointly. This dependence does not necessarily bias the pooled point estimate within an individual window, because each independent comparison contributed no more than one effect estimate to that window. However, treating the window-specific estimates as independent in cross-window comparisons would ignore their covariance and overstate the amount of independent information. As a result, the uncertainty of between-window contrasts or temporal-trend analyses could be under- or overestimated, potentially exaggerating or obscuring an apparent temporal pattern and making confidence intervals or P values from such comparisons unreliable. Differences in effect magnitude or statistical significance across windows should therefore not be interpreted as evidence that the treatment effect changed over time. In particular, the favorable pooled estimate within 24 h after surgery does not establish that the anxiolytic effect is confined to this period, and the available data do not determine how long it persists.

### 4.3 Clinical and methodological explanations for heterogeneity

Between-study differences in effect estimates may be related to variation in patient and surgical characteristics, anesthesia techniques, dexmedetomidine regimens, and methods of anxiety assessment. Although the prespecified subgroup test did not identify a statistically significant difference between saline and active-comparator studies, the small number of studies and wide confidence intervals limit the informativeness of this interaction test. Importantly, when saline-controlled comparisons were excluded in a sensitivity analysis, the point estimate remained in the direction of lower anxiety scores with dexmedetomidine, but the confidence interval included the null. Therefore, the present evidence should not be interpreted as demonstrating that dexmedetomidine is superior to active sedative or anxiolytic comparators. Substantial heterogeneity also persisted among active-comparator studies, indicating that comparator type alone did not explain the observed between-study variation. In exploratory analyses, a larger average effect was observed among studies using general anesthesia, and estimates also varied across anxiety measurement instruments, suggesting that the anesthesia context and measurement method may contribute to between-study variation. Because these study characteristics often varied concurrently, the findings are best regarded as indications that the effect may be context dependent rather than as a basis for defining specific patient populations likely to benefit (16).

Taken together, the findings for overall anxiety, anxiety within 24 h postoperatively, and postoperative sleep quality suggest that the clinical relevance of dexmedetomidine may lie in its combined perioperative effects. In settings where dexmedetomidine is already being considered as a sedative or anesthetic adjunct, reductions in early postoperative anxiety and improvements in postoperative sleep may be incorporated into treatment decisions as potential additional benefits. At the same time, the prediction intervals for both overall anxiety and anxiety within 24 h postoperatively crossed the null, indicating that the magnitude and direction of the anxiolytic effect may vary according to patient, surgical, anesthetic, and dosing characteristics. The pooled estimates therefore represent average effects across heterogeneous clinical settings (18). In addition, because SMDs are not expressed in the units of the original scales, the current findings cannot directly establish whether the observed changes in anxiety scores reached the clinically important difference for any specific instrument. Future studies should further evaluate the influence of dexmedetomidine regimen, baseline anxiety level, and assessment instrument on treatment effects within more homogeneous surgical and anesthetic settings.

### 4.4 Secondary outcomes and recovery implications

The sleep findings extend the clinical relevance of the primary anxiolytic findings. Consistent with a previous perioperative meta-analysis (53), these findings suggest that the potential benefits of dexmedetomidine may encompass both early postoperative anxiety and sleep quality. Improvements in sleep and reductions in early anxiety were directionally consistent; however, the available evidence is insufficient to establish a causal relationship between them or to determine whether improved sleep mediates the reduction in anxiety.

Neither depression nor pain outcomes showed a clear between-group difference. Each analysis included only 4 studies, with limited numbers of studies and participants. In addition, the pain studies varied substantially in surgical procedures, dexmedetomidine regimens, and assessment time points. These factors may have reduced the precision of the effect estimates and limited their interpretation. Accordingly, the available evidence is insufficient to determine the effects of dexmedetomidine on postoperative depression and pain, and larger, more consistently designed randomized controlled trials are needed.

These secondary outcomes suggest that the clinical value of dexmedetomidine is better understood in terms of its overall adjunctive role in perioperative care, rather than as a basis for expanding its indications according to any single outcome. Potential benefits involving anxiety, sleep, analgesia, and opioid sparing should be weighed against potential hemodynamic adverse effects, particularly bradycardia and hypotension (13). Among trials that provided explicit categorical event counts, bradycardia was reported in approximately 9% of dexmedetomidine-treated patients and 6% of control patients, whereas hypotension was reported in approximately 14% and 13%, respectively. These crude frequencies are descriptive rather than pooled comparative estimates, because event definitions, monitoring periods, and reporting methods varied substantially across studies. Moreover, information on the clinical severity of these events and the need for therapeutic intervention was inconsistently reported. Consequently, the available evidence does not permit a reliable estimate of the comparative risk of clinically significant bradycardia or hypotension, and incomplete and non-standardized safety reporting remains an important evidence gap.

### 4.5 Limitations

This study has several limitations. First, the number of included studies was limited, and study characteristics—including patient and surgical factors, anesthesia techniques, dexmedetomidine regimens, comparators, and anxiety measurement instruments—often varied concurrently. The available subgroup analyses therefore could not distinguish the independent contribution of each factor to differences in treatment effects. Accordingly, these findings primarily indicate potential context-specific variation and are insufficient to define the patient populations most likely to benefit or to identify the optimal dosing regimen. Second, the specific assessment time points differed across studies within the prespecified time windows, and some studies reported repeated measurements from the same participants across multiple windows. Consequently, the estimates for the different time windows were not fully independent. The time-window analyses may describe patterns of effect across perioperative stages but should not be used for formal comparisons of temporal trends or to determine the specific time point at which the effect ends (54). Third, anxiety was assessed primarily using subjective scales, and some assessments were conducted after dexmedetomidine administration or intraoperatively. Sedation-related changes in arousal and responsiveness may therefore have affected the interpretation of anxiety scores. Because the included studies did not consistently report sedation depth at the time of anxiety assessment, we could not quantify this influence. The use of multiple anxiety instruments also limited the translation of standardized effect estimates into clinically meaningful changes on specific scales. Fourth, in some studies, means and standard deviations were estimated from medians and interquartile ranges. After these studies were excluded, the direction of the primary findings remained unchanged, although substantial between-study heterogeneity persisted. In the leave-one-out analysis, the confidence interval crossed the null after either of 2 studies was excluded, suggesting that the direction of effect was generally stable but that the precision of the estimate was somewhat sensitive to individual studies and data-handling methods. Fifth, although the funnel plot and Pustejovsky–Rodgers test provided no evidence of small-study effects, publication bias and selective reporting could not be excluded given the limited number of studies and substantial heterogeneity (17). Sixth, only English-language publications were included, which may have resulted in the omission of a small amount of relevant evidence. The depression and pain analyses included few studies, which limited the precision and interpretability of these secondary outcomes. Safety outcomes were reported incompletely and non-standardly across trials, with substantial variation in event definitions, monitoring periods, and reporting of intervention requirements; consequently, although descriptive event frequencies could be summarized, the comparative risk of clinically significant adverse events could not be reliably estimated. These limitations constrained the interpretation of the secondary outcomes and the overall benefit–risk balance.

## 5 Conclusion

Perioperative dexmedetomidine was associated with reduced anxiety in adult surgical patients. Although the evidence for this association was clearer within 24 h after surgery, the limited data across time windows do not establish that the anxiolytic effect is confined to this period. These findings suggest a possible adjunctive role for dexmedetomidine in perioperative anesthetic management. Future well-designed stratified trials should determine which dexmedetomidine regimens provide the greatest benefit to particular patient populations and further clarify the underlying mechanisms.

## Data Availability

The original contributions presented in the study are included in the article/**Supplementary Material**. Further inquiries can be directed to the corresponding author.

## References

[1] Bello CM Eisler P Heidegger T . Perioperative anxiety: Current status and future perspectives. J Clin Med. (2025) 14:1422. doi: 10.3390/jcm14051422 40094863 PMC11901026

[2] Gümüs K . The effects of preoperative and postoperative anxiety on the quality of recovery in patients undergoing abdominal surgery. J Perianesth Nurs. (2021) 36:174–8. doi: 10.1016/j.jopan.2020.08.016 33640291

[3] Bedaso A Mekonnen N Duko B . Prevalence and factors associated with preoperative anxiety among patients undergoing surgery in low-income and middle-income countries: A systematic review and meta-analysis. BMJ Open. (2022) 12:e058187. doi: 10.1136/bmjopen-2021-058187 35277412 PMC8919464

[4] Friedrich S Reis S Meybohm P Kranke P . Preoperative anxiety. Curr Opin Anaesthesiol. (2022) 35:674–8. doi: 10.1097/aco.0000000000001186 36131642

[5] Gertler R Brown HC Mitchell DH Silvius EN . Dexmedetomidine: A novel sedative-analgesic agent. Proc (Bayl Univ Med Cent). (2001) 14:13–21. doi: 10.1080/08998280.2001.11927725 16369581 PMC1291306

[6] Mahmoud M Mason KP . Dexmedetomidine: Review, update, and future considerations of paediatric perioperative and periprocedural applications and limitations. Br J Anaesth. (2015) 115:171–82. doi: 10.1093/bja/aev226 26170346

[7] Silva-Jr JM Katayama HT Nogueira FAM Moura TB Alves TL de Oliveira BW . Comparison of dexmedetomidine and benzodiazepine for intraoperative sedation in elderly patients: A randomized clinical trial. Reg Anesth Pain Med. (2019) 44:319–24. doi: 10.1136/rapm-2018-100120 30777901

[8] Janse van Rensburg E Indiveri L Mogane P . The perioperative use of dexmedetomidine in paediatric patients. Children (Basel). (2025) 12:690. doi: 10.3390/children12060690 40564648 PMC12191122

[9] Wegner GRM Wegner BFM Cumming HS de Oliveira HG Campos LR da Silva LHD . Optimizing pediatric premedication for general anesthesia: A comprehensive Bayesian network meta-analysis. J Clin Anesth. (2025) 105:111903. doi: 10.1016/j.jclinane.2025.111903 40517567

[10] Yang WY Huang K Lin ZJ Zeng W Liu X Liu HB . Intranasal dexmedetomidine for the management of preoperative anxiety-related insomnia: A randomized, three-blinded, clinical trial compared with lorazepam and placebo. Drug Des Devel Ther. (2024) 18:6061–73. doi: 10.2147/dddt.S487463 39717197 PMC11663697

[11] Hao X Zhang Z Yang L Guo Y Cao F Cao J . Association of dexmedetomidine with postoperative depressive symptoms in older surgical patients: A prospective multicenter study. CNS Neurosci Ther. (2025) 31:e70407. doi: 10.1111/cns.70407 40387522 PMC12087293

[12] Hao X Zhang Z Cai C Yang L Cao F Chen G . Effect of intraoperative dexmedetomidine on postoperative mental health in cardiac surgery: A randomized controlled trial. Int J Surg. (2026) 112:3709–19. doi: 10.1097/js9.0000000000003906 41376475

[13] Weerink MAS Struys M Hannivoort LN Barends CRM Absalom AR Colin P . Clinical pharmacokinetics and pharmacodynamics of dexmedetomidine. Clin Pharmacokinet. (2017) 56:893–913. doi: 10.1007/s40262-017-0507-7 28105598 PMC5511603

[14] Page MJ McKenzie JE Bossuyt PM Boutron I Hoffmann TC Mulrow CD . The Prisma 2020 statement: An updated guideline for reporting systematic reviews. Bmj. (2021) 372:n71. doi: 10.1136/bmj.n71 33782057 PMC8005924

[15] Wan X Wang W Liu J Tong T . Estimating the sample mean and standard deviation from the sample size, median, range and/or interquartile range. BMC Med Res Methodol. (2014) 14:135. doi: 10.1186/1471-2288-14-135 25524443 PMC4383202

[16] Higgins JPT Thomas J Chandler J Cumpston M Li T Page MJ . editors. Cochrane Handbook for Systematic Reviews of Interventions Version 6.5 (2024), Updated August 2024 ed: Cochrane.

[17] IntHout J Ioannidis JP Borm GF . The Hartung-Knapp-Sidik-Jonkman method for random effects meta-analysis is straightforward and considerably outperforms the standard Dersimonian-Laird method. BMC Med Res Methodol. (2014) 14:25. doi: 10.1186/1471-2288-14-25 24548571 PMC4015721

[18] Riley RD Higgins JP Deeks JJ . Interpretation of random effects meta-analyses. Bmj. (2011) 342:d549. doi: 10.1136/bmj.d549 21310794

[19] Pustejovsky JE Rodgers MA . Testing for funnel plot asymmetry of standardized mean differences. Res Synth Methods. (2019) 10:57–71. doi: 10.1002/jrsm.1332 30506832

[20] Etemadi SM Kaviani N Salimian K Tajmiri G . Effect of dexmedetomidine added to lidocaine cartridge on the level of patient sedation, cooperation, and patient and surgeon satisfaction during mandibular third-molar extraction surgery: A randomized double-blind controlled trial. Int J Dent. (2022) 2022:4722674. doi: 10.1155/2022/4722674 36193181 PMC9525784

[21] Goettel N Bharadwaj S Venkatraghavan L Mehta J Bernstein M Manninen PH . Dexmedetomidine vs propofol-remifentanil conscious sedation for awake craniotomy: A prospective randomized controlled trial. Br J Anaesth. (2016) 116:811–21. doi: 10.1093/bja/aew024 27099154

[22] Kang E Lee KH Jeon SY Lee KW Ko MJ Kim H . The timing of administration of intravenous dexmedetomidine during lower limb surgery: A randomized controlled trial. BMC Anesthesiol. (2016) 16:116. doi: 10.1186/s12871-016-0282-2 27871236 PMC5117579

[23] Karaaslan D Peker TT Alaca A Ozmen S Kirdemir P Yorgancigil H . Comparison of buccal and intramuscular dexmedetomidine premedication for arthroscopic knee surgery. J Clin Anesth. (2006) 18:589–93. doi: 10.1016/j.jclinane.2006.03.019 17175428

[24] Kim HJ Lee D Ri HS Choi J Choi J Rhee SJ . Objective assessment of perioperative anxiety using functional near-infrared spectroscopy in elderly patients: A prospective randomized observational pilot study. Int J Med Sci. (2023) 20:1763–73. doi: 10.7150/ijms.89287 37928873 PMC10620860

[25] Shi H Yang D Liu J . Intranasal dexmedetomidine in termination of first trimester pregnancy of suction evacuation. Asian J Anesthesiol. (2018) 56(1):1–13. doi: 10.1016/j.aja.2017.09.001 29847967

[26] Sun Y Liu C Zhang Y Luo B She S Xu L . Low-dose intramuscular dexmedetomidine as premedication: A randomized controlled trial. Med Sci Monit. (2014) 20:2714–9. doi: 10.12659/msm.891051 25529851 PMC4278696

[27] Wang Y Fang X Liu C Ma X Song Y Yan M . Impact of intraoperative infusion and postoperative PCIA of dexmedetomidine on early breastfeeding after elective cesarean section: A randomized double-blind controlled trial. Drug Des Devel Ther. (2020) 14:1083–93. doi: 10.2147/dddt.S241153 32210537 PMC7073426

[28] Wu LP Kang WQ . Effect of dexmedetomidine for sedation and cognitive function in patients with preoperative anxiety undergoing carotid artery stenting. J Int Med Res. (2020) 48:300060520938959. doi: 10.1177/0300060520938959 32972265 PMC7522831

[29] Xiong J Gao J Pang Y Zhou Y Sun Y Sun Y . Dexmedetomidine premedication increases preoperative sedation and inhibits stress induced by tracheal intubation in adult: A prospective randomized double-blind clinical study. BMC Anesthesiol. (2022) 22:398. doi: 10.1186/s12871-022-01930-z 36544098 PMC9768986

[30] Xu Z Zhang L Liu Y Meng Y Ling Z Yuan S . Effect of perioperative intravenous infusion of dexmedetomidine on the quality of early and long-term postoperative recovery in patients undergoing thoracoscopic surgery: A randomized controlled trial. Minerva Anestesiol. (2025) 91:621–30. doi: 10.23736/s0375-9393.25.18716-6 40338293

[31] Yu C Li S Deng F Yao Y Qian L . Comparison of dexmedetomidine/fentanyl with midazolam/fentanyl combination for sedation and analgesia during tooth extraction. Int J Oral Maxillofac Surg. (2014) 43:1148–53. doi: 10.1016/j.ijom.2014.03.019 24794760

[32] Zhang L Hao LJ Hou XL Wu YL Jing LS Sun RN . Preoperative anxiety and postoperative pain in patients with laparoscopic hysterectomy. Front Psychol. (2021) 12:727250. doi: 10.3389/fpsyg.2021.727250 34707538 PMC8542785

[33] Zhang L Zhang W Duan C Hao L Jing L Hou X . Analysis of the impact of dexmedetomidine on the vital signs of patients with anxiety at various time intervals upon entering the operating room. BMC Psychiatry. (2025) 25:663. doi: 10.1186/s12888-025-07107-5 40597954 PMC12219959

[34] Xiao-Hong C Meng-Meng L Huang H Yi-Qing H Yi-Nuo Q Ming-Zi R . Effect of dexmedetomidine on perioperative anxiety in thoracic surgery patients. Med J Chin People's Liberation Army. (2022) 47:464–70. doi: 10.11855/j.issn.0577-7402.2022.05.0464

[35] Yao J Shen Z Jin H Ma T Wang J Li S . Dexmedetomidine after deep brain stimulation for prevention of delirium in elderly patients with Parkinson's disease: Protocol for a single-centre, randomised, double-blind, placebo-controlled trial in China. BMJ Open. (2023) 13:e070185. doi: 10.1136/bmjopen-2022-070185 37433729 PMC10347446

[36] Fu W Xu H Zhao T Xu J Wang F . Effects of dexmedetomidine combined with etomidate on postoperative cognitive function in older patients undergoing total intravenous anaesthesia: A randomized, double-blind, controlled trial. BMC Geriatr. (2024) 24:97. doi: 10.1186/s12877-024-04726-7 38273248 PMC10809642

[37] Gu X Tan X Chen J Wang J Lu Y Zhang L . The clinical effect of dexmedetomidine combined with parecoxib sodium on sedation, antianxiety and prevention of intubation stress in patients undergoing functional endoscopic sinus surgery: A randomised controlled trial. BMC Anesthesiol. (2020) 20:166. doi: 10.1186/s12871-020-01080-0 32631301 PMC7336422

[38] He J Zhang X Li C Fu B Huang Y Li H . Dexmedetomidine nasal administration improves perioperative sleep quality and neurocognitive deficits in elderly patients undergoing general anesthesia. BMC Anesthesiol. (2024) 24:42. doi: 10.1186/s12871-024-02417-9 38291398 PMC10826024

[39] Jouybar R Kazemifar S Asmarian N Karami A Khademi S . Comparison of the effect of melatonin, dexmedetomidine, and gabapentin on reduction of postoperative pain and anxiety following laminectomy: A randomized clinical trial. BMC Anesthesiol. (2022) 22:318. doi: 10.1186/s12871-022-01851-x 36243712 PMC9569101

[40] Kulkarni AM Gurav SY Shibupaulose L . Anaesthetic and haemodynamic effects of dexmedetomidine vs midazolam used as premedication in minor obstetrics and gynaecological procedures- a prospective interventional study. J Clin Diagn Res. (2022) 16:UC20–4. doi: 10.7860/JCDR/2022/56374.16460

[41] Li J Zhou YH Yang YJ Wang Q Lei W Li SX . Effect of dexmedetomidine for epidural supplementation on postpartum sleep disturbance after cesarean delivery: A double-blind, randomized clinical trial. Int J Surg. (2025) 111:4495–507. doi: 10.1097/js9.0000000000002489 40391965

[42] Lim YP Yahya N Izaham A Kamaruzaman E Zainuddin MZ Wan Mat WR . The comparison between propofol and dexmedetomidine infusion on perioperative anxiety during regional anesthesia. Turk J Med Sci. (2018) 48:1219–27. doi: 10.3906/sag-1802-126 30541250

[43] Mukherjee B Backiavathy V Sujatha R . A prospective randomized double-blinded study of dexmedetomidine versus propofol infusion for orbital surgeries. Saudi J Ophthalmol. (2020) 34:77–81. doi: 10.4103/1319-4534.305021 33575526 PMC7866727

[44] Wang F Xie D Xu H Ye Q Wu L Gao XP . The effects of remifentanil-propofol combined with dexmedetomidine on cognitive dysfunction in elderly patients after ureteroscopic holmium laser lithotripsy: A double-blind randomized controlled trial. Trials. (2022) 23:192. doi: 10.1186/s13063-022-06121-2 35241145 PMC8895834

[45] Yu Y Li Y Han D Gong C Wang L Li B . Effect of dexmedetomidine on posttraumatic stress disorder in patients undergoing emergency trauma surgery: A randomized clinical trial. JAMA Netw Open. (2023) 6:e2318611. doi: 10.1001/jamanetworkopen.2023.18611 37326991 PMC10276303

[46] Zhang L He Y Chen L Liu X Zhang T Zhou X . Low-dose esketamine plus dexmedetomidine in patient-controlled intravenous analgesia improves post-cesarean sleep quality: A double-blind randomized trial. Drug Des Devel Ther. (2025) 19:9789–96. doi: 10.2147/dddt.S556396 41209150 PMC12595983

[47] Zi J Fan Y Dong C Zhao Y Li D Tan Q . Anxiety administrated by dexmedetomidine to prevent new-onset of postoperative atrial fibrillation in patients undergoing off-pump coronary artery bypass graft. Int Heart J. (2020) 61:263–72. doi: 10.1536/ihj.19-132 32132317

[48] Wang K Wu M Xu J Wu C Zhang B Wang G . Effects of dexmedetomidine on perioperative stress, inflammation, and immune function: Systematic review and meta-analysis. Br J Anaesth. (2019) 123:777–94. doi: 10.1016/j.bja.2019.07.027 31668347

[49] Qiu G Wang P Rao J Qing X Cao C Wang D . Dexmedetomidine inhibits paraventricular corticotropin-releasing hormone neurons that attenuate acute stress-induced anxiety-like behavior in mice. Anesthesiology. (2024) 140:1134–52. doi: 10.1097/aln.0000000000004982 38498811

[50] Jiang L Zhao J Xia M Song Y Cao H Wang Y . Dexmedetomidine elicits a prolonged anxiolytic effect by inhibiting adrenergic neurons in the locus coeruleus in mice. Transl Psychiatry. (2025) 15:487. doi: 10.1038/s41398-025-03682-z 41253759 PMC12627444

[51] Wang L Liang XQ Sun YX Hua Z Wang DX . Effect of perioperative dexmedetomidine on sleep quality in adult patients after noncardiac surgery: A systematic review and meta-analysis of randomized trials. PloS One. (2024) 19:e0314814. doi: 10.1371/journal.pone.0314814 39636906 PMC11620464

[52] Ben Simon E Rossi A Harvey AG Walker MP . Overanxious and underslept. Nat Hum Behav. (2020) 4:100–10. doi: 10.1038/s41562-019-0754-8 31685950

[53] Liu H Wei H Qian S Liu J Xu W Luo X . Effects of dexmedetomidine on postoperative sleep quality: A systematic review and meta-analysis of randomized controlled trials. BMC Anesthesiol. (2023) 23:88. doi: 10.1186/s12871-023-02048-6 36944937 PMC10029163

[54] Trikalinos TA Olkin I . Meta-analysis of effect sizes reported at multiple time points: A multivariate approach. Clin Trials. (2012) 9:610–20. doi: 10.1177/1740774512453218 22872546

