# Supplementary material for: The anxiolytic effect of dexmedetomidine in adult perioperative patients: a systematic review with meta-analysis of randomized controlled trials
Source: Front Psychiatry. 2026 Sep 15;17:1957118. doi: 10.3389/fpsyt.2026.1957118 (PMC13619925; doi:10.3389/fpsyt.2026.1957118)
Supplement: Supplementary file 1 [file Supplementaryfile1.docx]

Supplementary Material

# 1 eMethod: search strategy

Embase:

| **#** | **Searches** | **Results** |
| --- | --- | --- |
| 1 | Dexmedetomidine/ | 6575 |
| 2 | (Dexmedetomidine or Dexmedetomidine Hydrochloride or Hydrochloride Dexmedetomidine or MPV-1440 or mir1440 or MPV 1440 or preceded or igalme or sepadex or silex or cepedea or deudor or dexmonitor).ab,ti,kw. | 68241 |
| 3 | Anxiety/ | 126575 |
| 4 | (Anxiety or Angst or Nervousness or Hypervigilance or Social Anxiety or Anxieties or Social Anxieties or Anxiousness).ab,ti,kw. | 332620 |
| 5 | (Perioperative or postoperative or Preoperative or Intraoperative).ab,ti,kw. | 1035730 |
| 6 | 1 or 2 | 68483 |
| 7 | 3 or 4 | 357555 |
| 8 | 5 and 6 and 7 | 195 |

Pubmed:

(((((((((((((((Dexmedetomidine[Title/Abstract]) OR (Dexmedetomidine Hydrochloride[Title/Abstract])) OR (Hydrochloride, Dexmedetomidine[Title/Abstract])) OR (MPV-1440[Title/Abstract])) OR (MPV1440[Title/Abstract])) OR (MPV 1440[Title/Abstract])) OR (Precedex[Title/Abstract])) OR (Igalmi[Title/Abstract])) OR (Sedadex[Title/Abstract])) OR (Sileo[Title/Abstract])) OR (Cepedex[Title/Abstract])) OR (Dexdor[Title/Abstract])) OR (Dexdomito[Title/Abstract])) AND (((((((((Anxiety[Title/Abstract]) OR (Angst[Title/Abstract])) OR (Nervousness[Title/Abstract])) OR (Hypervigilance[Title/Abstract])) OR (Social Anxiety[Title/Abstract])) OR (Anxieties, Social[Title/Abstract])) OR (Anxiety, Social[Title/Abstract])) OR (Social Anxieties[Title/Abstract])) OR (Anxiousness[Title/Abstract]))) AND ((((Perioperative) OR (postoperative)) OR (Preoperative)) OR (Intraoperative))) AND (((("Randomized Controlled Trial" ) OR ("Randomized Controlled Trials as Topic")))))

Web of Science:

Dexmedetomidine OR Dexmedetomidine Hydrochloride OR Hydrochloride Dexmedetomidine OR MPV-1440 OR mir1440 OR MPV 1440 OR preceded OR igalme OR sepadex OR silex OR cepedea OR deudor OR dexmonitor

Anxiety OR Angst OR Nervousness OR Hypervigilance OR Social Anxiety OR Anxieties OR Social Anxieties OR Anxiousness

Randomized Controlled Trial OR Randomized Controlled Trials as Topic

Perioperative OR postoperative OR Preoperative OR Intraoperative

Cochrane:

#1	MeSH descriptor: [Dexmedetomidine] explode all trees

#2 (Dexmedetomidine or Dexmedetomidine Hydrochloride or Hydrochloride, Dexmedetomidine or MPV-1440 or MPV1440 or MPV 1440 or Precedex or Igalmi or Sedadex or Sileo or Cepedex or Dexdor or Dexdomitor):ti,ab,kw

#3 #1 or #2

#4 MeSH descriptor: [Anxiety] explode all trees

#5 (Anxiety or Angst or Nervousness or Hypervigilance or Social Anxiety or Anxieties, Social or Anxiety, Social or Social Anxieties or Anxiousness):ti,ab,kw

#6 #4 or #5

#7 MeSH descriptor: [Perioperative Period] explode all trees

#8 (Perioperative or postoperative or Preoperative or Intraoperative):ti,ab,kw

#9 #7 or #8

#10 #3 and #6 and #9

Medline:

(Dexmedetomidine OR Dexmedetomidine Hydrochloride OR Hydrochloride Dexmedetomidine OR MPV-1440 OR mir1440 OR MPV 1440 OR preceded OR igalme OR sepadex OR silex OR cepedea OR deudor OR dexmonitor).ab,ti,kw.

(Anxiety OR Angst OR Nervousness OR Hypervigilance OR Social Anxiety OR Anxieties OR Social Anxieties OR Anxiousness) .ab,ti,kw.

(Perioperative OR postoperative OR Preoperative OR Intraoperative).ab,ti,kw.

# 2 Supplementary Figures and Tables

**Supplementary Table 1 GRADE certainty of evidence assessment for each outcome.**

| **Outcome** | **Risk of bias** | **Inconsistency** | **Indirectness** | **Imprecision** | **Publication bias** | **Effect estimate (95% CI)** | **Participants (studies)** | **Conclusion** | **Certainty of evidence** |
| --- | --- | --- | --- | --- | --- | --- | --- | --- | --- |
| **Overall anxiety score** **(all prespecified perioperative time points)** | Not serious | Serious | Not serious | Serious | Not detected | SMD -0.73 (-1.37 to -0.10) I²=94.2%; 95% PI -2.97 to 1.50 | 1,373 (12 RCTs) | Dexmedetomidine probably lowers perioperative anxiety scores, although the magnitude varies substantially across settings. | **⊕⊕◯◯** **Low** |
| **Anxiety score within 24 h after surgery** | Not serious | Serious | Not serious | Serious | Not detected | SMD -0.83 (-1.58 to -0.07) I²=92.9%; 95% PI -2.76 to 1.11 | 841 (6 RCTs) | Dexmedetomidine probably lowers anxiety scores within 24 h; the effect size is heterogeneous. | **⊕⊕◯◯** **Low** |
| **Early postoperative sleep score** **(higher score consistently coded as worse)** | Not serious | Not serious | Not serious | Not serious | Not detected | SMD -0.64 (-0.81 to -0.48) I²=0%; 95% PI -0.81 to -0.48 | 620 (4 RCTs) | Dexmedetomidine improves early postoperative sleep scores. | **⊕⊕⊕⊕** **High** |
| **Postoperative depression score** **(24 h to 42 d)** | Not serious | Not serious | Not serious | Serious | Not detected | SMD -0.25 (-0.51 to 0.00) I²=0% | 437 (4 RCTs) | Dexmedetomidine probably produces a small reduction in depression scores, but the interval reaches no effect. | **⊕⊕⊕◯** **Moderate** |
| **Pain score** **(intraoperative to 25 h postoperative; 0-10 scale)** | Not serious | Serious | Not serious | Serious | Not detected | MD -1.02 points (-2.11 to 0.08) I²=84.6%; 95% PI -3.36 to 1.33 | 549 (4 RCTs) | Dexmedetomidine may reduce pain scores, but the true effect may range from important benefit to no effect. | **⊕⊕◯◯** **Low** |
| **Safety** **(bradycardia, hypotension, respiratory depression and other adverse events)** | Serious | Serious | Not serious | Very serious | Not detected | Not pooled; definitions, monitoring windows and denominators differed | Up to 1,413 (12 RCTs) | The evidence is very uncertain about overall safety; non-reporting must not be interpreted as absence of adverse events. | **⊕◯◯◯** **Very low** |

Supplementary eTable 1 Summary of findings and certainty of evidence for perioperative dexmedetomidine compared with control interventions in adult surgical patients. The certainty of evidence was assessed using the Grading of Recommendations Assessment, Development and Evaluation approach across the domains of risk of bias, inconsistency, indirectness, imprecision, and publication bias. Outcomes included overall perioperative anxiety, anxiety within 24 h after surgery, postoperative sleep quality, depression, pain, safety outcomes. CI, confidence interval; DEX, dexmedetomidine; GRADE, Grading of Recommendations Assessment, Development and Evaluation; MD, mean difference; PI, prediction interval; RCT, randomized controlled trial; SMD, standardized mean difference.

**Supplementary Table 2 Study-level descriptive safety reporting for bradycardia, hypotension, and other cardiovascular findings.**

| **Study** | **Comparison and sample size** | **Bradycardia** | **Hypotension** | **Other cardiovascular findings / reporting limitation** |
| --- | --- | --- | --- | --- |
| Kulkarni et al. 2022 | DEX 30 vs midazolam 30 | 1/30 vs 0/30; the DEX patient received atropine | MAP <60 mmHg prespecified; counts NR | HR, SBP, DBP, and MAP decreased more with DEX but remained within the reported acceptable range. |
| Zi et al. 2020 | DEX 62 vs propofol 61 | 3/62 vs 1/61 | 3/62 vs 3/61 | POAF: 10/62 vs 20/61 (P=0.037). |
| Gu et al. 2020 | D vs N and DP vs P; n=30/group | NR as a separate outcome | No significant hypotension stated; counts NR | Arrhythmia: D 1/30 vs N 3/30; DP 0/30 vs P 1/30. |
| Lim et al. 2018 | DEX 42 vs propofol 42 | NR | NR | HR and MAP decreased in both groups; MAP reduction persisted longer with DEX. |
| Jouybar et al. 2022 | DEX, gabapentin, or melatonin; n=33/group | NR | NR | No between-group difference in vital-sign changes (P=0.451); categorical events were not reported. |
| He et al. 2024 | Intranasal DEX 40 vs saline 40 | 2/40 vs 2/40 | 1/40 vs 3/40 | Hypertension: 2/40 vs 7/40. |
| Li et al. 2025 | Epidural DEX 60 vs control 60 | 0/60 vs 0/60 | 6/60 vs 6/60 | Tachycardia: 3/60 vs 5/60; hypertension: 1/60 vs 0/60. |
| Yu et al. 2023 | DEX 156 vs saline 154 | 28/156 vs 20/154 (P=0.23) | 39/156 vs 32/154 (P=0.38) | Tachycardia: 11 vs 21; hypertension: 5 vs 10; postoperative stroke, MI, and HF: 0 in both groups. |
| Fu et al. 2024 | DEX 66 vs control 66; two cohorts combined | NR | NR | Only serial SBP, DBP, and HR were reported; no categorical event counts. |
| Wang et al. 2022 | DEX 60 vs saline 60 | NR | NR | Treatment thresholds were HR <50 bpm and SBP <90 mmHg; no patient required rescue treatment. |
| Zhang et al. 2025 | Esketamine+DEX 35 vs esketamine 35; basic control 35 | 0/35 vs 0/35; basic control 0/35 | 0/35 vs 0/35; basic control 0/35 | NR |
| Mukherjee et al. 2020 | DEX 10 vs propofol 10 | No events/complications reported | No events/complications reported | MAP decreased in both groups; HR decreased significantly only with propofol. |

NR, not reported; DEX, dexmedetomidine; D/DP/N/P, trial-group labels; HR, heart rate; SBP, systolic blood pressure; DBP, diastolic blood pressure; MAP, mean arterial pressure; POAF, postoperative atrial fibrillation; MI, myocardial infarction; HF, heart failure.

## 2.1 Supplementary Figures

Supplementary Figure 1. Subgroup analysis of anxiety scores according to comparator type. (The total estimate is based on the subgroup analytic dataset and is not equivalent to the primary overall estimate.).

Supplementary Figure 2. Leave-one-out sensitivity analysis restricted to active-comparator studies after exclusion of saline-controlled comparisons.

Supplementary Figure 3. Subgroup analysis of anxiety scores by anaesthesia type.

**Supplementary Figure 4.** Subgroup analysis of anxiety scores by assessment instrument.

**Supplementary Figure 5.** Forest plot of postoperative depression scores.

**Supplementary Figure 6.** Forest plot of postoperative pain scores.

**Supplementary Figure 7.** Forest plot of postoperative sleep quality. Negative values indicate better sleep quality with dexmedetomidine.

**Supplementary Figure 8.** Leave-one-out sensitivity analysis of overall anxiety scores.

**Supplementary Figure 9.** Sensitivity analysis of overall anxiety scores after excluding studies requiring data conversion.

**Supplementary Figure10.** Sensitivity analysis of anxiety scores within 24 h after surgery after excluding studies requiring data conversion.

**Supplementary Figure11.** Sensitivity analysis of anxiety scores 24–72 h after surgery after excluding studies requiring data conversion.

**Supplementary Figure 12.** Sensitivity analysis by comparator type after excluding studies requiring data conversion.

**Supplementary Figure13.** Sensitivity analysis by anaesthesia type after excluding studies requiring data conversion.

**Supplementary Figure 14.** Sensitivity analysis by anxiety assessment instrument after excluding studies requiring data conversion.

**Supplementary Figure 15.** Sensitivity analysis of postoperative depression scores after excluding studies requiring data conversion.

**Supplementary Figure 16.** Sensitivity analysis of postoperative pain scores after excluding studies requiring data conversion.

**Supplementary Figure 17.** Sensitivity analysis of postoperative sleep quality after excluding studies requiring data conversion. Negative values indicate better sleep quality with dexmedetomidine.

Supplementary Figure 18. Contour-enhanced sample-size funnel plot for overall anxiety scores.
